# Biochemical Characterization of *Aspergillus fumigatus* AroH, a Putative Aromatic Amino Acid Aminotransferase

**DOI:** 10.3389/fmolb.2018.00104

**Published:** 2018-11-28

**Authors:** Mirco Dindo, Egidia Costanzi, Marco Pieroni, Claudio Costantini, Giannamaria Annunziato, Agostino Bruno, Nancy P. Keller, Luigina Romani, Teresa Zelante, Barbara Cellini

**Affiliations:** ^1^Department of Experimental Medicine, University of Perugia, Perugia, Italy; ^2^P4T group, Department of Food and Drug, University of Parma, Parma, Italy; ^3^Experimental Therapeutics Program, IFOM—The FIRC Institute for Molecular Oncology Foundation, Milan, Italy; ^4^Department of Medical Microbiology and Immunology, Department of Bacteriology, University of Wisconsin, Madison, WI, United States

**Keywords:** pyridoxal 5'-phosphate, aromatic amino acid aminotransferase, fungal infection, homology modeling, enzyme spectroscopy, enzyme kinetics, enzymatic assay

## Abstract

The rise in the frequency of nosocomial infections is becoming a major problem for public health, in particular in immunocompromised patients. *Aspergillus fumigatus* is an opportunistic fungus normally present in the environment directly responsible for lethal invasive infections. Recent results suggest that the metabolic pathways related to amino acid metabolism can regulate the fungus-host interaction and that an important role is played by enzymes involved in the catabolism of L-tryptophan. In particular, in *A. fumigatus* L-tryptophan regulates *Aro* genes. Among them, AroH encodes a putative pyridoxal 5'-phosphate-dependent aminotransferase. Here we analyzed the biochemical features of recombinant purified AroH by spectroscopic and kinetic analyses corroborated by *in silico* studies. We found that the protein is dimeric and tightly binds the coenzyme forming a deprotonated internal aldimine in equilibrium with a protonated ketoenamine form. By setting up a new rapid assay method, we measured the kinetic parameters for the overall transamination of substrates and we demonstrated that AroH behaves as an aromatic amino acid aminotransferase, but also accepts L-kynurenine and α-aminoadipate as amino donors. Interestingly, computational approaches showed that the predicted overall fold and active site topology of the protein are similar to those of its yeast ortholog, albeit with some differences in the regions at the entrance of the active site, which could possibly influence substrate specificity. Should targeting fungal metabolic adaptation be of therapeutic value, the results of the present study may pave the way to the design of specific AroH modulators as potential novel agents at the host/fungus interface.

## Introduction

Health care-associated, or nosocomial infections is a rising threat in public health. According to the World Health Organization, nosocomial infections affect 14% of admitted patients worldwide (Suleyman and Alangaden, 2016). The increase of opportunistic or nosocomial fungal infections has been perceived as a consequence of advances and more aggressive treatment modalities as organ transplantation, stem cell transplantation, immunomodulatory molecules or sophisticated chemotherapy. These conditions now represent high risk factors for invasive fungal infections. In particular, *Aspergillus* spp. are ubiquitous saprophytic molds that thrive on a diverse array of organic matter. These fungi are inhaled as spores daily without triggering any particular infectious disease, thus underlining the non-pathogenic potential of the fungus, unless severe immunocompromised hosts are exposed to spores. The most common site of infection is the lung, where either neutropenia or neutrophilia may destabilize the mucosal lung homeostasis and thus contribute to high fungal infection burden (Romani et al., 2008; Choera et al., 2017). Nevertheless, recent studies highlight the importance of regulatory cells in the defense against the fungus, as fungus-specific T regulatory cells have been described in healthy individuals (Bacher et al., 2016). This implies that resistance to fungal infections requires a very sophisticated but balanced environment where immune metabolism represents an important aspect (Choera et al., 2017). Host metabolic regulation in mammals may strongly regulate the outcome of fungal infections (Romani et al., 2008, 2009). However, one of the factors associated with the peculiar virulence of the fungus is its metabolic versatility through which the fungus increases the capacity to growth and adapt to stress conditions (Kwon-Chung and Sugui, 2013). Thus, targeting metabolic reprogramming in the fungus could be exploited for therapeutic intervention.

Immune tolerance in fungal infections is triggered by the enzyme indoleamine 2,3-dioxygenase (IDO)1 that pivotally regulates L-tryptophan metabolism. Consistent with the complex metabolic dynamics in terms of tryptophan catabolism in pathogens (Wang et al., 2016; Choera et al., 2017), L-tryptophan metabolism in *Aspergillus* is regulated by several pathways (Choera et al., 2017). In order to identify proteins possibly involved in L-tryptophan degradation, a BLAST analysis on *Saccharomyces cerevisiae* Genome Database (www.yeastgenome.org) was performed and allowed the identification of two classes of genes, *aro* and *ido* (Choera et al., 2017). Both gene sets were found up-regulated in response to tryptophan in *Aspergillus* (Wang et al., 2016). While the *Aspergillus ido* genes are well characterized (Yuasa and Ball, 2012), little is known about the expression and function of the *aro* genes (Wang et al., 2016).

*Ido* genes (*idoA* Afu3g14250, *idoB* Afu4g09830, *idoC* Afu7g02010) encode putative indoleamine 2,3-dioxygenases which dioxygenate L-tryptophan to produce L-kynurenine (Wang et al., 2016). Among the *aro* genes, *aroH* (Afu2g13630) encodes a putative pyridoxal 5'-phosphate (PLP)-dependent aromatic aminotransferase, which transaminates L-tryptophan to generate indolepyruvate. AroH shows the highest sequence identity (~50%) with Aro8 of *S. cerevisiae* as well as with Aro8 recently characterized in *Candida albicans*, an opportunistic yeast often diagnosed in immunocompromised hosts (Rao et al., 2010; Rzad et al., 2018). *C. albicans* Aro8 is definitely the most versatile enzyme among the aminotransferases already investigated. It may catabolize L-histidine, L-lysine, and aromatic amino acids. Yeast Aro8 is a broad specificity dimeric aminotransferase belonging to the Fold Type I family of PLP-enzymes (Bulfer et al., 2013), able to metabolize both aromatic amino acids and α-aminoadipate (Iraqui et al., 1998). It has been recently claimed as one of the enzymes involved in L-tryptophan degradation (Ohashi et al., 2017). A detailed study on the main genes playing a role in *A. fumigatus* virulence, does not include Aro proteins (Abad et al., 2010). Nevertheless, it is widely accepted that the catalytic versatility of aminotransferases plays a key role in the adaptation and survival of pathogenic fungi in the changing environment of the host by allowing the growth using different nitrogen sources (Rzad et al., 2018). Therefore, fungal aminotransferases may constitute a basis for the nutritional flexibility in the fungus *Aspergillus*, and the characterization of Aros enzymes may be pivotal for drug design of innovative therapies targeting fungal metabolic check points.

The objective of the present work was to study the biochemical properties of recombinant purified *Aspergillus fumigatus* AroH, by combining spectroscopic, kinetic and bioinformatic analyses. The results obtained not only shed light on the functional and structural features of this enzyme, but will also provide the necessary base to design and test small-molecule ligands acting as specific inhibitors.

## Materials and methods

### Materials

The plasmid AroHhis, which contains the entire AroH cDNA along with a C-terminal 6xHis tag cloned in the BamHI/NotI sites of a pET22b prokaryotic expression vector, was purchased from GenScript (New Jersey, USA). PLP, L-tryptophan, L-tyrosine, L-phenylalanine, α-aminoadipate, α-ketoglutarate, α-ketoadipate, L-glutamate dehydrogenase from bovine liver (GDH), 3-acetylpyridine adenine dinucleotide (APAD^+^), L-lactic dehydrogenase (LDH), and isopropyl-β-D-thiogalactopyranoside, were purchased from Sigma. All other chemicals were of the highest purity available.

### Expression and purification of recombinant AroH

We transformed *E.coli* BL21(DE3) cells with the vector AroHhis. We grew transformed cells in Luria-Bertani broth at 37°C to a turbidity of 0.4–0.6 at 600 nm and then induced AroH expression by 0.3 mM isopropyl-β-D-thiogalactopyranoside in the presence of 50 μM PLP for 15 h at 30°C. The presence of PLP during induction was found to increase protein yields in preliminary experiments, possibly as a consequence of its ability to enter bacterial cells and behave as a chaperone (Rodionov et al., 2009; Cellini et al., 2014). Cells were harvested and resuspended in 20 mM sodium phosphate buffer pH 7.4, 0.5 M NaCl containing 20 mM imidazole, 100 μM PLP, and Protease inhibitors cocktail (Sigma). Cell lysis was obtained by addition of 0.2 μg/ml lysozyme and incubation for 15 min at room temperature, followed by a freeze-thaw cycle (Dindo et al., 2016). The suspension was centrifuged at 30,000 *g* for 30 min at 4°C and the obtained lysate diluted to about 20 mg/ml was then loaded on a HisPrep FF 16/10 column (GE Healthcare) equilibrated with 20 mM sodium phosphate buffer pH 7.4 containing 0.5 M NaCl and 20 mM imidazole. A linear gradient with the same buffer containing 500 mM imidazole (0–100% in 200 ml) allowed to elute AroH between 250 and 300 mM imidazole. After addition of 100 μM PLP, the protein solution was subjected to forced dialysis with 100 mM potassium phosphate buffer (KP), pH 7.4 using Amicon Ultra 10 concentrators (Millipore) to remove imidazole and unbound coenzyme. The stock protein, at a concentration of 200 μM can be stored at −20°C without loss of activity for at least 1 month. The apparent molar absorption coefficient at 280 nm of the AroH monomer was determined as described by Pace (Pace et al., 1995) and found to be 84503 M^−1^ cm^−1^.

### SDS-PAGE and western-blot analyses

Ten micrograms of cell lysate or chromatography eluted fractions were loaded per lane on a 10% SDS-PAGE gel along with the Precision plus protein Kaleidoscope™ (Bio-Rad) molecular mass markers. For Western-blot analyses, the proteins were transferred on a nitrocellulose membrane and the membrane was blocked in 5% milk for 1 h at 37°C. Recombinant AroH detection was obtained by incubating the membrane with a 6x-His Tag Monoclonal Antibody HRP (Thermo Fisher; dilution 1:2000) for 15 h at 4°C and then washing three times in TBST (50 mM Tris-HCl pH 7.5, 150 mM NaCl, 0.1% Tween 20). Blotted proteins were detected with ECL^®;^ (Millipore), using the ChemiDoc XRS Imaging System (Bio-Rad, Hercules, CA).

### Size-exclusion chromatography (SEC)

The molecular dimensions of holoAroH were determined by loading the protein at 5 μM concentration on a Superdex 200 10/300 GL column equilibrated and run with 0.1 M KP pH 7.4 on a Akta Start FPLC system (GE Healthcare). The injection volume was 150 μl at a flow rate of 0.5 ml/min with detection at 280 nm. The elution volume of each peak was compared to that of a set of molecular weight standards under the same experimental conditions, to calculate the apparent hydrodynamic radius of the protein.

### Preparation of AroH in the apoform

HoloAroH (10–20 μM) was incubated with 20 mM L-tryptophan for 20 min at room temperature in a final volume of 1 ml. The reaction mixture was then subjected to forced dialysis using Amicon Ultra 4 devices (Millipore), washed twice with 1 M KP, pH 6, and then 4–5 times with 0.1 M KP, pH 7.4, as previously described (Cellini et al., 2007).

### Measurement of the equilibrium dissociation constant for PLP (k_D(PLP)_)

ApoAroH (0.1 μM) was incubated in the presence of various PLP concentrations (0.02–10 μM) in 0.1 M KP pH 7.4. After 15 h incubation, the intrinsic fluorescence emission spectrum of each sample was registered and the quenching of the intrinsic fluorescence was calculated. The K_D(PLP)_ was obtained using a tight-binding model according to the following equation:

(1) $$\begin{array}{r} Y=Y_{\max}\;\frac{\begin{array}{c} \left[\text{E}\right]\text{t}+\left[\text{PLP}\right]\text{t }+K_{D\left(PLP\right)}-\sqrt{{\left[\text{E}\right]\text{t}+\left[\text{PLP}\right]\text{t }+K_{D\left(PLP\right)}}^{2}-4\left[\text{E}\right]\text{t}\left[\text{PLP}\right]\text{t }} \end{array}}{2\left[\text{E}\right]\text{t}} \end{array}$$

where [E]_t_ and [PLP] represent the total concentration of dimeric AroH and PLP, respectively, Y refers to the intrinsic fluorescence quenching at the PLP concentration [PLP], and Y_max_ refers to the intrinsic fluorescence quenching when all enzyme molecules are complexed with coenzyme (Montioli et al., 2015).

### Enzyme assays

The transaminase activity of recombinant purified AroH in the presence of various substrates was measured by a coupled assay with GDH. L-glutamate formation was determined by monitoring its conversion to α-ketoglutarate and the concomitant reduction of APAD^+^, which gives rise to an increase in the absorbance at 365 nm. Assays were performed at 25°C on a Jasco V-750 spectrophotometer (Jasco Europe S.r.l.). A typical reaction mixture contained 10–20 mM amino acid substrate, 1 mM α-ketoglutarate, 2 mM APAD^+^, 1.5 mg/ml of GDH in 66 mM KP, pH 8.2, in a final volume of 300 μl. The reaction was initiated by addition a mixture of PLP (100 μM) with 0.1–0.5 μM of AroH. One unit of activity was defined as the amount of enzyme needed to convert 1 nmol of APAD^+^ to APADH per second at 25°C. A molar extinction coefficient of 9,100 M^−1^ cm^−1^ for APADH formation at 365 nm was used. The determination of the kinetic parameters of AroH for aromatic amino acids was performed by measuring the initial velocity of the transamination reaction at varying concentrations of each substrate in the presence of 1 mM α-ketoglutarate. In the case of α-ketoglutarate, the assays have been performed using 20 mM L-tryptophan as amino donor and varying the amino acceptor concentration from 0.025 to 1 mM, i.e., at concentrations below the limit for GDH inhibition.

The kinetic parameters for L-glutamate, L-kynurenine, α-aminoadipate, and α-ketoadipate were determined by a previously published discontinuous assay (Rzad and Gabriel, 2015). The reaction was carried out in 66 mM KP pH 7.4 in the presence of 100 μM PLP and AroH (0.2–0.5 μM) in a total volume of 100 μl. The reaction was terminated by heating the samples at 100°C for 2 min. In the case of L-glutamate and α-ketoadipate, 5 or 10 μl of the reaction mixture were added to 270 μl of 50 mM KP pH 7.4 containing 0.05 M NH_4_Cl, 0.5 mM NADH, 1.5 mg/ml GDH. Formation of NAD^+^ was measured spectrophotometrically by the decrease of the absorbance at 340 nm using the ε_M_ = 6200 dm^3^ cm^−1^ mol^−1^. In the case of L-kynurenine and α-aminoadipate, L-glutamate production was determined by adding 10 μl of the reaction mixture to 270 μl of 66 mM KP pH 8.3 containing GDH (1.5 mg/ml) and 2 mM APAD^+^. Formation of APADH was measured spectrophotometrically by the increase of the absorbance at 365 nm using the ε_M_ = 9100 dm^3^ cm^−1^ mol^−1^.

Initial velocity data obtained at varying substrate concentrations were fitted to the Michaelis-Menten equation using the software Origin 8.0 (OriginLab Corporation, Northampton, MA).

### Determination of optimum pH and melting temperature

To determine the optimum pH, AroH enzymatic activity was measured at 25°C in the pH range 6.0–8.3 in 0.1 M KP by the spectrophotometric assay described above using L-phenylalanine or L-tryptophan and α-ketoglutarate as substrates. The thermal stability of AroH in the apo and holo-form was determined by using two different approaches: (1) the holoenzyme in the presence of 20 μM PLP or the apoenzyme, both at a concentration of 10 μM, were preincubated for 10 min at different temperatures ranging from 25 to 85°C. Then, 100 μM PLP was added to the solution of apoAroH, and samples were placed on ice for 15 min. Samples were assayed for transaminase activity at 25°C as described above; (2) the thermal unfolding of holo- and apoAroH, at a concentration of 1 μM, was monitored by following the loss of tertiary structure using the intrinsic fluorescence emission signal at 330 and 332 nm or the internal aldimine emission signal at 420 nm of the holo-form, in 0.1 M KP, pH 7.4, with temperature increasing of 0.5 °C/min from 20 to 90°C. Data were fitted to a least square method present on the Melting analysis of the Spectra manager software (Jasco Europe S.r.l.).

### Spectroscopic measurements

Absorption spectra were recorded on a Jasco V-750 spectrophotometer (Jasco Europe S.r.l.). The enzyme solutions were filtered through a 0.22 μM filter to reduce light scattering from the small amount of precipitate. Fluorescence spectra were taken by a Jasco FP-8200 spectrofluorimeter (Jasco Europe S.r.l.) using 5 nm excitation and emission bandwidths at a protein concentration of 1 μM. Spectra of blanks, i.e., of samples containing all components except AroH, were taken immediately prior to measurements of samples containing enzyme, and subtracted from spectra of the samples. CD spectra were obtained by a Jasco J-710 spectropolarimeter using a thermostatically controlled cell compartment at 25°C. For near-UV and visible wavelengths, a protein concentration of 4 μM was used in a cuvette with a 1 cm path length. For far-UV measurements, the protein concentration was 1 μM with a path length of 0.1 cm. Routinely, four spectra were recorded at a scan speed of 50 nm/min with a bandwidth of 2 nm and averaged automatically except where indicated. Secondary-structure content was calculated from far-UV CD spectra using the CD spectra deconvolution software Dichroweb as previously reported (Cellini et al., 2007).

The effect of pH on the AroH absorption and fluorescence spectra was evaluated by resuspending the protein in solutions of KP buffered at different pHs ranging from 6.02 to 8.31. The pK_spec_ was determined by fitting the values of absorbance or fluorescence intensity as a function of pH to the equation for a sigmoidal curve.

### Homology model construction and validation

The primary sequence of AroH was obtained from the National Center for Biotechnology Information (NCBI) database in FASTA format. The AroH structural model was built using the SWISS-MODEL portal (https://swissmodel.expasy.org) (Waterhouse et al., 2018), based on a target-template alignment. Upon searching the template library using BLAST (Camacho et al., 2009) and HHBlits (Remmert et al., 2011), the crystal structure of Aro8 from *Saccharomyces cerevisiae* (PDB accession: 4JE5) was chosen as template (Bulfer et al., 2013). The global quality of the modeled structure was evaluated based on the root-mean-square deviation (RMSD) value, obtained by overlapping the peptide chain of the conserved regions of the generated model using UCSF Chimera (Pettersen et al., 2004). The validation of the stereochemical quality of the model was determined by both the Ramachandran plot, which was generated using PROCHECK (Laskowski et al., 1993), and scores provided by ERRAT (Colovos and Yeates, 1993) and Verify3D (Bowie et al., 1991). Additional structural evaluations and stereo chemical analyses were performed using the ProSA-web server [https://prosa.services.came.sbg.ac.at/prosa.php; Wiederstein and Sippl, 2007 and QMEAN (Benkert et al., 2011)] on the SWISS-MODEL portal. These tools correlate the scores obtained for various parameters of the constructed model, including local geometry, distance-dependent interaction potential, and solvation potential, with the corresponding scores of reference structures experimentally determined by X-ray crystallography.

### Electrostatic analysis of protein surface

The electrostatic potential maps of Aro8 and AroH were calculated by the adaptive Poisson-Boltzmann solver 1.3 tool, using the non-linear Poisson–Boltzmann equation, as previously reported (Dindo et al., 2017). The graphical interface of the tool is integrated in UCSF CHIMERA version 1.1 (Pettersen et al., 2004). The calculations were performed on the Opal webserver. Structure preparation and analysis of the protonation state of residues at different pH values were carried out by the PDB2PQR ((Dolinsky et al., 2004); using CHARMM as force field) and the PROPKA 3.0 (Olsson et al., 2011) tools, respectively, on the Opal web service (Ren et al., 2010).

## Results and discussion

### AroH expression and purification

**Figure 1:**
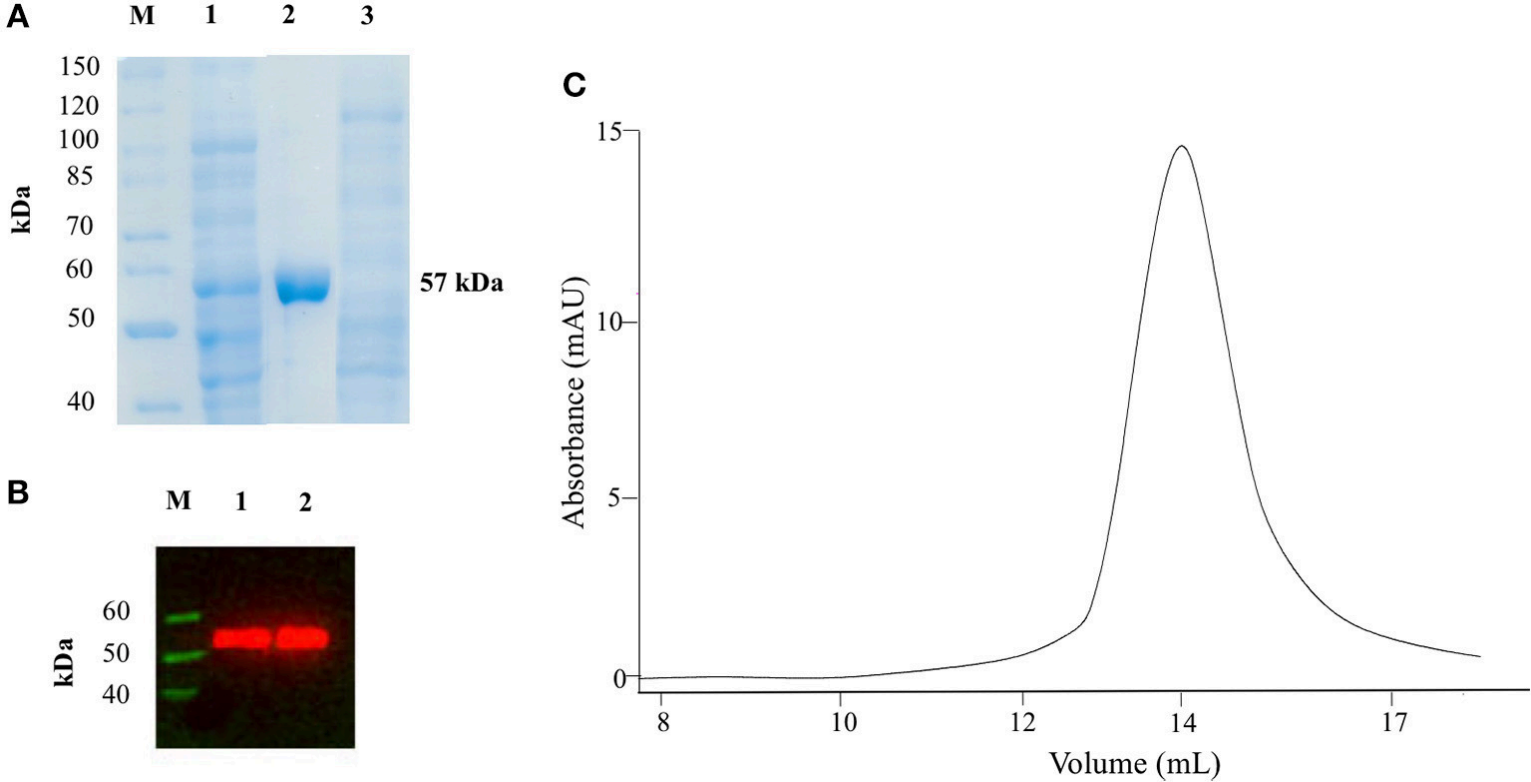
SDS-PAGE, Western Blot and SEC analyses on recombinant AroH. **(A)** SDS-PAGE of recombinant AroH purification. Lane M: protein molecular weight markers; lane 1: 30 μg of total bacterial lysate upon induction with IPTG; lane 2: 20 μg of purified recombinant AroH after affinity chromatography; lane 3: 30 μg of column flow-through. **(B)** Western blot of purified AroH. Lane M: molecular weight marker of His-tagged proteins; lane 1: 10 μg of total lysate after induction with IPTG; lane 2: 5 μg of purified recombinant AroH. **(C**) Elution profile of 5μM AroH on a Superdex 200 SEC column equilibrated and run in 0.1M KP pH 7.4.

We expressed AroH fused with a C-terminal 6xHis tag in *E. coli* BL21 cells transformed with the plasmid pET22b and purified the protein by affinity chromatography. The yield was 12.5 mg of pure protein per liter of bacterial culture. Recombinant purified AroH was more than 95% pure, as estimated by SDS-PAGE, migrated with an apparent size of 57 kDa, in line with the expected subunit mass of 59480 Da deduced from the amino acid sequence, and bound to an antibody against the histidine tag (Figures 1A,B). The protein eluted from SEC with a symmetric peak at 14.1 ml (Figure 1C) corresponding to an apparent MW of 107 kDa, thus indicating that it is a homodimer, as previously observed for the orthologs Aro8 from *Saccaromyces cerevisiae* and *Candida albicans* (Karsten et al., 2011; Rzad and Gabriel, 2015). Moreover, it bound 1 mol of PLP per mol of subunit, as determined by releasing the coenzyme in 0.1 M NaOH.

### Spectral features of purified AroH

**Figure 2:**
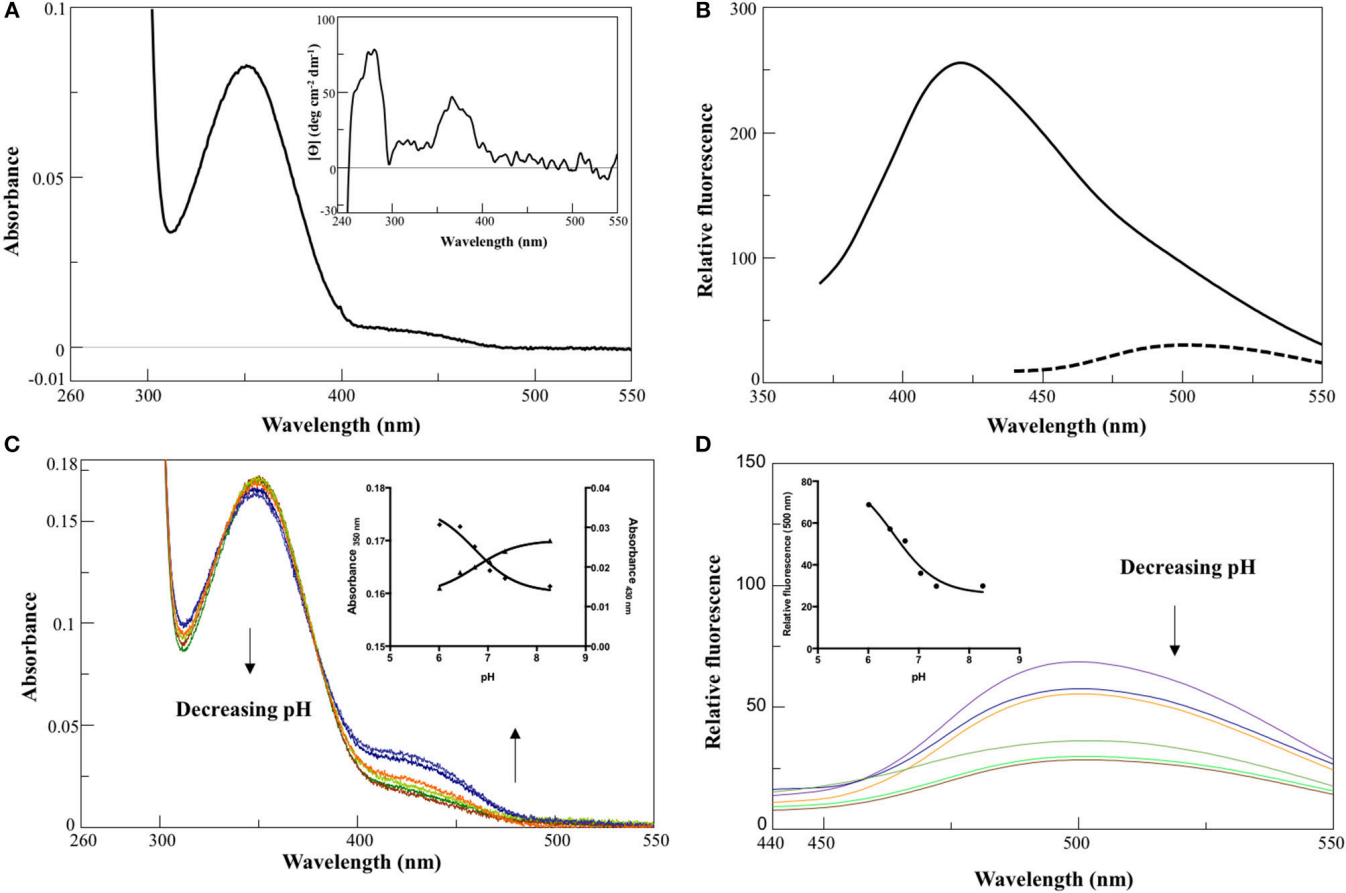
Spectroscopic features of holoAroH. (**A)** Absorbance and CD (inset) spectrum of 8 μM holoAroH in 0.1 M KP, pH 7.4. **(B)** Internal aldimine emission fluorescence of 1 μM holoAroH upon excitation at 350 nm (—) and 432 nm (- -). **(C,D)** pH-dependence of the visible absorbance spectrum of holoAroH (10 μM) and of the internal aldimine emission fluorescence at 500 nm (exc. at 432 nm; 1 μM enzyme concentration) in KP 0.1 M at pH 6.02, 6.43, 6.75, 7.03, 7.44, and 8.31. The insets of both panels show the fitting of data to obtain the pK_spec_.

In 0.1 M KP pH 7.4, the visible absorbance spectrum of AroH in the holo-form displayed a main band at 350 nm, with an Abs_280_/Abs_350_ ratio of 8, and a shoulder around 432 nm (Figure 2A), due to PLP bound to Lys348. The band at 350 nm corresponded to a positive dichroic band at 365 nm (inset of Figure 2A). Positive bands in the 260–280 region were also visible in the CD spectrum, and they are probably related to aromatic residues located in the vicinity of the enzyme active site. Upon excitation at 350 nm, an emission spectrum was obtained with a maximum at 420 nm and a shoulder around 500 nm, while the excitation at 432 nm gave a band at 500 nm (Figure 2B), typical of Schiff bases of PLP with amines (Honikel and Madsen, 1972). Therefore, we attributed the peak at 350 nm to the unprotonated form of the internal aldimine, and the shoulder at 432 nm to the ketoenamine tautomer of the protonated aldimine. By lowering the pH from 8.3 down to 6.0, we observed limited spectral changes consisting in the reduction in the intensity of the 350 nm band accompanied by a slight red-shift and by the concomitant increase of the absorbance at 432 nm (Figure 2C). Similarly, an increased emission of the protonated internal aldimine at 500 nm (excitation at 432 nm) was observed at decreasing pH (Figure 2D). Upon fitting the absorbance changes as a function of pH we obtained pK_spec_ values of the internal aldimine equal to 6.7 ± 0.1 and 6.7 ± 0.2 at 350 and 432 nm, respectively (inset of Figure 2C). Analogous results were obtained by fitting the change of the internal aldimine emission fluorescence as a function of pH (pK_spec_ = 6.5 ± 0.1). Overall, these spectroscopic features are in line with those already reported for other PLP-enzymes of the same family (Hayashi et al., 1998; Karsten et al., 2011; Okada et al., 2012; Campanini et al., 2013), and suggest that the AroH active site presents a similar active site microenvironment.

**Figure 3:**
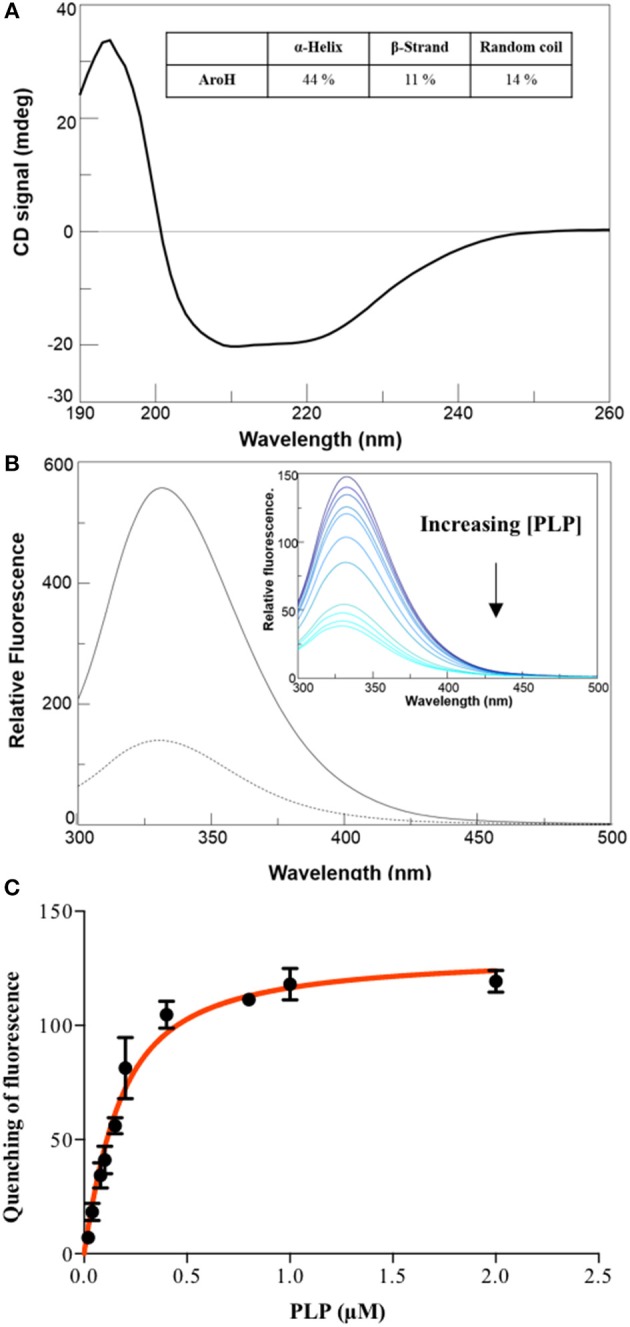
Secondary and tertiary structure of AroH in the apo and holo-form**. (A)** Far-UV CD spectrum registered at 1 μM enzyme concentration in KP 0.1 M pH 7.4. The inset shows the percentage of secondary structure obtained by spectrum deconvolution. **(B)** Intrinsic fluorescence emission (exc. at 280 nm) of apo- (—) and holo- (- -) AroH at 0.5 μM concentration in KP 0.1 M pH 7.4. The inset shows the quenching of intrinsic emission fluorescence at increasing PLP concentrations. **(C**) K_D(PLP)_ calculation. The graph shows the quenching of the AroH intrinsic fluorescence as a function of PLP concentration. The line shows the theoretical fit obtained using Equation 1.

The far-UV CD spectrum of recombinant purified holoAroH displayed two minima at 208 and 222 nm, typical of proteins with a high α-helix content (Figure 3A). The deconvolution of the spectrum gave the secondary structure composition reported in the inset of Figure 3A. The values are similar to those available from the structure of Aro8 from yeast (Karsten et al., 2011), thus confirming the predicted similarity between the two proteins. Upon excitation at 280 nm, the emission spectrum of holoAroH showed a maximum at 330 nm, mainly due to tryptophan residues buried in a hydrophobic environment in the folded protein (Figure 3B).

By treating holoAroH with an excess of L-tryptophan and subsequently removing the formed pyridoxamine 5'-phosphate (PMP) by forced dialysis, we generated the apo-form of the protein. ApoAroH did not display any absorbance signal in the visible region, but reconstitution with PLP led to the complete recovery of the catalytic activity and of the spectral features of the holoenzyme. As shown in Figure 3B, the intrinsic fluorescence emission spectrum of apoAroH showed a 2 nm blue-shifted maximum and an intensity about 3-fold higher with respect to the holo-form (Figure 3B), thus suggesting that in the holoenzyme a quenching of the tryptophan emission fluorescence occurs. Upon incubation of apoAroH at increasing PLP concentration and determination of the percentage of intrinsic fluorescence quenching (inset of Figure 3B), we calculated a K_D(PLP)_ value for the enzyme-coenzyme complex equal to 0.10 ± 0.02 μM, which is indicative of a tight binding (Figure 3C). In fact, among the PLP dependent enzymes, it has been reported that transaminases show the highest affinity for the coenzyme (Jenkins and Fonda, 1985).

**Figure 4:**
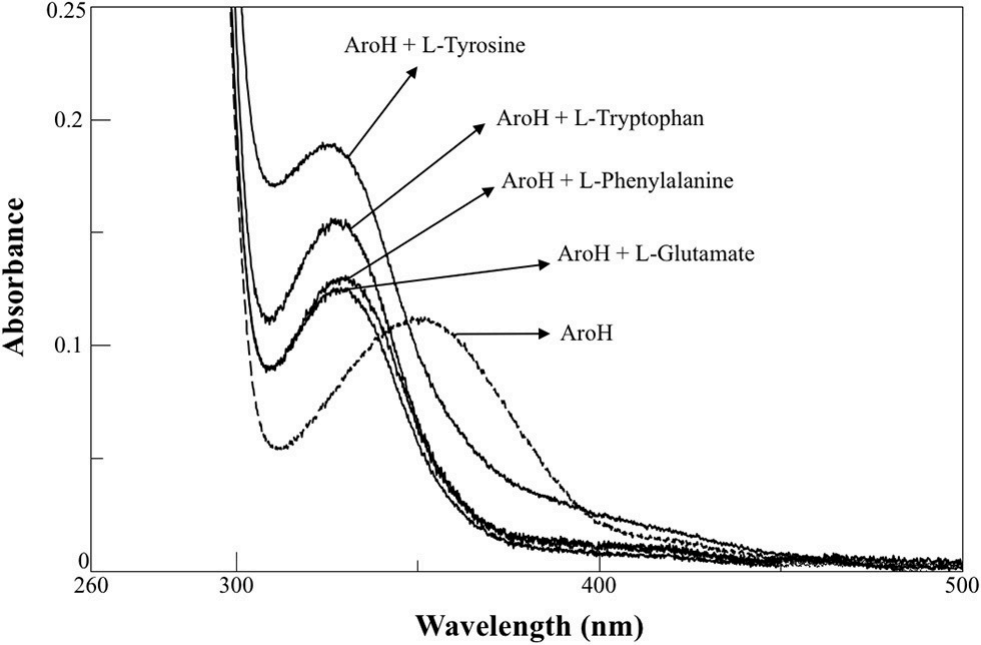
External aldimine of AroH upon binding with different amino donors. AroH (10 μM) (- - - ) was incubated with 6 mM L-tyrosine, 20 mM L-tryptophan, 15 mM L-phenylalanine, or 100 mM L-glutamate, as indicated (—), in 0.1 M KP pH 7.4, at 25°C.

AroH has been classified as an aromatic aminotransferase, based on its similarity with *S. cerevisiae* Aro8 (Choera et al., 2017). Moreover, the protein displays a significant sequence homology with human kynurenine aminotransferase II (hKATII), an enzyme that utilizes both kynurenine and α-aminoadipate as amino donors (Han et al., 2008; Campanini et al., 2013). Based on these findings, we examined the spectral changes occurring in holoAroH upon interaction with various amino acid substrates (Figure 4). Upon addition of L-tryptophan, L-phenylalanine, L-tyrosine or L-glutamate, we observed the disappearance of the band at 350 nm and of the shoulder at 420 nm and the concomitant appearance of a band centered at 325 nm, which represents the PMP form of the enzyme. Similar results were also obtained upon interaction with L-kynurenine and α-aminoadipate, although the high absorbance in the visible region of the first and the low solubility of the second compromised the obtainment of clear spectra (data not shown). Overall, these data suggest that aromatic amino acids as well as L-kynurenine and α-aminoadipate could behave as substrates for AroH.

### Setup of a new spectrophotometric assay for AroH

Aromatic aminotransferase assays are usually based on the measurement of the increase in the absorbance in the 300–350 nm region to monitor α-ketoacid formation. At high substrate concentrations, the sensitivity of these assays could be affected by the significant absorbance of the amino donor, while at low substrate concentrations the measured rate values could be affected by the occurrence of the reverse reaction due to the accumulation of the amino acid product. To overcome these limitations, we designed a new continuous assay to measure AroH transaminase activity by coupling it with GDH, which consumes produced L-glutamate with the concomitant formation of a reduced cofactor absorbing in the visible region. Since the equilibrium constant of GDH is shifted toward L-glutamate production, instead of NAD^+^ as hydrogen acceptor we used APAD^+^, a NAD^+^-analog endowed with a higher reduction potential. Moreover, we performed the reaction in 66 mM KP at pH 8.2, in the absence of ammonium ions, as required based on their inhibitory effect against GDH (Miñambres et al., 2000). As a preliminary analysis, we checked the reliability of the coupled reaction using known amounts of L-glutamate. Moreover, we determined the amount of coupled enzyme sufficient to avoid rate-limiting effects in the continuous assay. Finally, considering that GDH activity is affected by α-ketoglutarate, we checked the upper limit of α-ketoglutarate concentration to prevent inhibition effects, which resulted to be 1 mM (Holzer et al., 1963). Figure S1 shows a typical AroH assay curve (panel A) and the negative control curves in the absence of AroH or in the absence of APAD^+^ (panel B). As shown in the inset of Figure S1A, the assay is linear up to 0.5 μM enzyme concentration.

### Kinetic characterization of recombinant AroH

**Figure 5:**
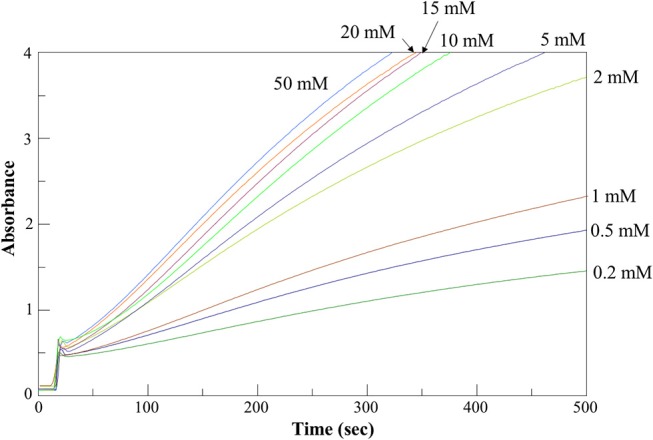
AroH L-phenylalanine transaminase activity assay. Time-dependent changes of the absorbance at 365 nm during the continuous assay of AroH (0.2 μM) transaminase activity in the presence of L-phenylalanine at the indicated concentrations and 1 mM α-ketoglutarate in 66 mM KP, pH 7.4 at 25°C.

We determined the kinetic parameters of the enzyme for the various substrates using α-ketoglutarate as amino acceptor, along with those for α-ketoadipate and α-ketoglutarate using L-glutamate and L-tryptophan, respectively, as amino donors (Table 1). Figure 5 shows representative assay curves obtained at various concentrations of L-phenylalanine, using α-ketoglutarate as amino acceptor.

**Table 1:** Steady-state kinetic parameters of AroH for the transamination reaction.

**Substrate**	**Co-substrate**	**K_M_ (mM)**	***k*_cat_ (s^−1^)**	***k*_cat_/K_M_** **(s^−1^ mM^−1^)**
L-tryptophan	α-ketoglutarate	2.1 ± 0.3	3.0 ± 0.1	1.4 ± 0.2
L-tyrosine	α-ketoglutarate	0.5 ± 0.1	4.0 ± 0.3	8 ± 2
L-phenylalanine	α-ketoglutarate	1.4 ± 0.2	7.4 ± 0.2	5.2 ± 0.6
L-glutamate^*^	α-ketoadipate	10 ± 1	10.3 ± 0.3	0.96 ± 0.08
L-kynurenine^*^	α-ketoglutarate	9 ± 2	4.1 ± 0.4	0.4 ± 0.1
α-aminoadipate^*^	α-ketoglutarate	3.3 ± 0.4	13.2 ± 0.5	4.0 ± 0.4
α-ketoglutarate	L-tryptophan	0.18 ± 0.02	2.5 ± 0.1	14 ± 2
α-ketoadipate^*^	L-glutamate	0.14 ± 0.03	12.5 ± 1.0	90 ± 20

* *Kinetic parameters determined by a discontinuous assay, as described in the Materials and Methods section*.

AroH displayed K_m_ and *k*_cat_ values of the same order of magnitude for the aromatic substrates, although L-tryptophan had a lower value of catalytic efficiency (driven by a 4-fold higher K_m_ and a 5-fold lower *k*_cat_) with respect to L-tyrosine, which resulted to be the best amino donor. The catalytic efficiency for α-aminoadipate was similar to that for aromatic amino acids, while those for L-kynurenine and L-glutamate were lower. Again, α-ketoadipate was a better amino acceptor with respect to α-ketoglutarate, mainly due to a higher *k*_cat_. Overall, these data confirm the placement of AroH in the class of aromatic aminotransferases, which are characterized by a broad substrate specificity but use aromatic and dicarboxylic amino acids as preferred substrates (Okamoto et al., 1998; Karsten et al., 2011). As compared with similar enzymes, AroH displays an analogous preference for L-tyrosine, L-phenylalanine and α-aminoadipate as amino donors (Karsten et al., 2011). We also demonstrated that it is able to metabolize L-kynurenine, as already suggested for Aro8 based on studies on knock-out yeast mutants (Ohashi et al., 2017) reporting that the enzyme is responsible for tryptophan catabolism to kynurenic acid. However, it should be pointed out that the K_m_ of L-kynurenine of AroH is 5 to 20-fold higher than that for aromatic substrates. Finally, the finding that AroH displays the highest catalytic efficiency toward α-ketoadipate suggests that the enzyme could be also involved in lysine metabolism, as recently reported for orthologs from *Candida albicans* (Rzad et al., 2018).

### Stability studies

**Figure 6:**
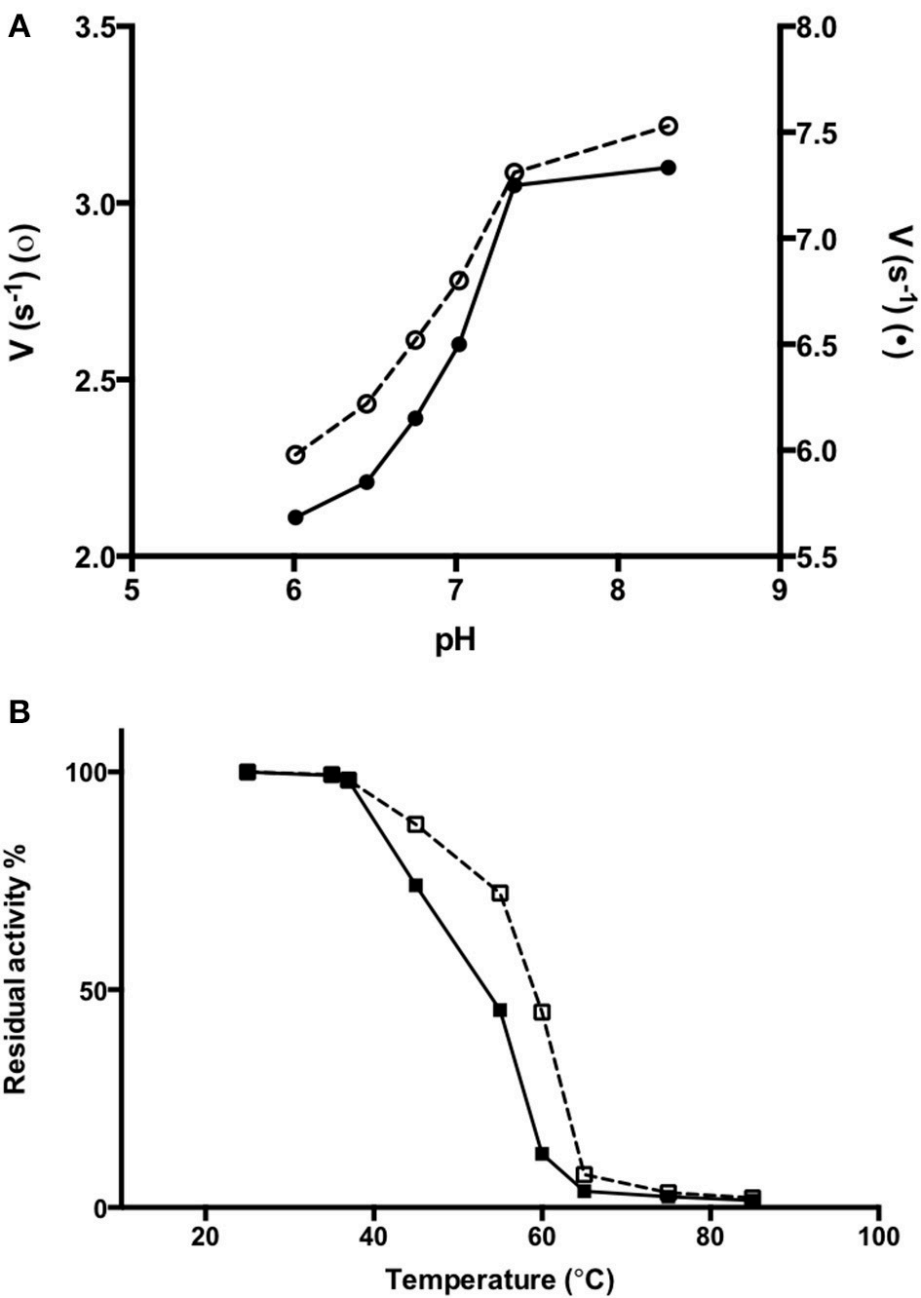
Activity of AroH as a function of pH and temperature. **(A)** AroH activity as a function of pH. The activity measurements were performed in KP 0.1 M at 25°C. The amino donor used were L-tryptophan (o) or L-phenylalanine (•) while the amino acceptor was α -ketoglutarate. The reaction was stopped in 10% TCA (v/v) after 10 min. L-glutamate determination was performed as reported in Material and Methods. **(B)** The figure shows the residual enzymatic activity of AroH in the holo-(□) and apo-(■) form in KP 0.1 M pH 7.4 at 25°C, upon 10 min incubation in the temperature range 25–85°C. In both panels the continuous and dashed line indicate the holo- or apo-form, respectively.

In order to obtain information about the biophysical properties of recombinant purified AroH, we determined the optimum pH for transaminase activity, using L-phenylalanine and α-ketoglutarate as substrates. As shown in Figure 6A, AroH exhibits its maximum catalytic activity at alkaline pH, in line with data reported on other fungal α-aminoadipate aminotransferases (Rzad and Gabriel, 2015).

We then tested the general stability of the protein by inducing the thermal unfolding and measuring the residual activity, the loss of tertiary structure and, in the case of the holo-form, coenzyme release (Figures 6B, S2). We found that holoAroH undergoes unfolding, accompanied by loss of coenzyme and enzymatic activity, with half-temperatures of approximately 60°C, while apoAroH undergoes thermal unfolding with half-temperatures of approximately 52°C (Table 2). These results confirm the stabilizing role of the coenzyme in Fold Type I PLP-dependent enzymes (see below for the model of the AroH structure), where the active site is formed by residues belonging to both subunits, and the binding of PLP should shift the equilibrium toward the formation of the dimer (Oppici et al., 2016).

**Table 2:** Half-temperatures of apo- and holoAroH unfolding.

	**Tm_unf(λexc 280 nm)_**	**Tm_PLP(λexc 350 nm)_**	**T_50(activity)_**
ApoAroH	52.6°C	–	52.1°C
HoloAroH	60.8°C	61.3°C	58.8°C

*The values of Tm_unf_ and Tm_PLP_ were calculated from the temperature-dependent loss of tertiary structure or bound coenzyme, respectively, using fluorescence spectroscopy. The values of T_50(activity)_ were calculated from the data of residual activity upon incubation a different temperatures*.

### Homology modeling of the AroH structure

As expected, the primary structure of AroH shows the best sequence alignment (42% similarity, 90% query coverage, e-value 8e-135, bit score 383) with Aro8 from *S. cerevisiae*, whose crystal structure is available (PDB id: 4JE5, resolution 1.9 Å) (Figure S3; Bulfer et al., 2013). Gaps between the template and query protein were found to represent 5% of the sequence. Therefore, we used the structure of Aro8 as template for the AroH homology modeling. It should be noted that 4 subunits generating two Aro8 dimers are present in the PDB file 4JE5. We modeled the structure of AroH using as target only the dimer formed by subunits A and B. In subunit A the PLP cofactor does not form a Schiff base linkage with Lys305, while in subunit B the coenzyme is present in the PMP form. Nevertheless, only in the AB dimer the interaction of the N-terminus with PLP can be appreciated, and thus it allows a more reliable prediction of the overall architecture of the AroH active site. In fact, it has been reported that the N-terminal region in proteins of the same family as AroH (such as hKATII and aminoadipate aminotransferase from *T. thermophilus*) is important for substrate channeling and thus could influence substrate specificity (Han et al., 2008; Bulfer et al., 2013).

**Figure 7:**
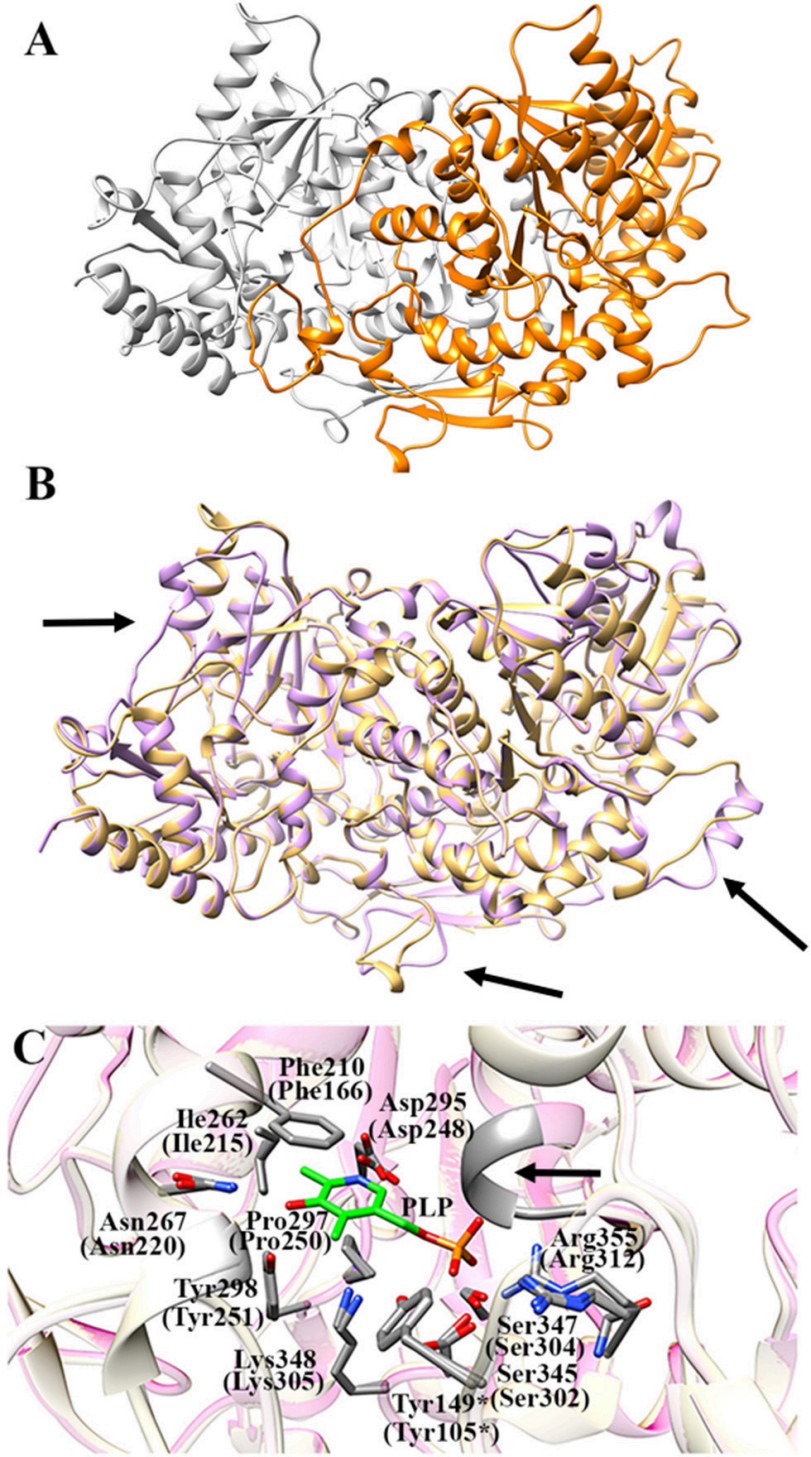
AroH homology model**. (A)** Homology model of dimeric AroH as ribbon representation obtained using the SWISS-MODEL portal. The two monomers are colored orange and white. **(B)** Superimposition between the structure of Aro8 (light purple) and that modeled for AroH (light orange). Flexible regions are indicated with arrows. **(C)** Active site topology of superimposed AroH and Aro8 structures. Residues involved in PLP binding are represented as gray sticks. The PLP cofactor is represented as green stick. The arrow indicates the position of the main-chain of Ser185 and Asn186. Tyr149^*^ belongs to the neighboring subunit. Numbers under parentheses indicate residue position in Aro8.

Figure 7A shows the obtained AroH homology model. The evaluation of the quality of the model is reported in Figure S4. We found a QMEAN Z-score of −1.69, and a z-score of −9.85 on the ProSA web server, values similar to those of structures of proteins determined by X-ray crystallography. In addition, all residues had a negative score, thus confirming model reliability. The profile-3D score of the homology model was 90.32, meaning that 90.32% of the residues had an average 3D-1D score ≥2. Using the ERRAT protein verification server, which analyzes the statistics of non-bonded interactions between different atom types, we found a score for the AroH model of 85.52, implying that the backbone conformation and non-bonded interactions of the model were acceptable. Finally, the Ramachandran plot ϕ/ψ distribution of backbone conformation angles for each residue revealed that 92.1% amino acids were in favored regions, 7.6% were in allowed (or generously allowed) regions and 0.2% in the outlier region.

It should be noted that a stretch of 50 amino acids present at the N-terminus of AroH is lacking in the Aro8 primary structure, and thus was excluded from the model. This region is not predicted to contain strong subcellular targeting sequences, although its role as a signal possibly dictating the localization of the protein in a specific intracellular compartment cannot be excluded. Further investigations will be necessary to shed light on the role of this region for the structural and/or functional properties of the protein.

The superimposition of the AroH model over its template structure of *S. cerevisiae* Aro8 (PDB id: 4JE5: Figure 7B) resulted in a backbone root mean square deviation (RMSD) of 1.23 Å which is considered of good quality being lower than 2 Å and confirms that the optimized model is satisfactory. We only noticed (i) the presence of a region that in the structure of Aro8 forms a α-helix (residues 263-272), while in the model of AroH is predicted to form a loop (residues 308-317); (ii) a different arrangement of a loop located on the surface of the two proteins (residues 131–141 in AroH, residues 84–94 in Aro8); (iii) the presence of a longer loop in the 435–465 region of AroH with respect to the corresponding one in Aro8 (residues 369–500) (Figure 7B).

Notably, the modeled AroH structure and Aro8 share the same residues (although at different positions in the sequence) that are located at the active site and involved in PLP binding (Figure 7C). The PLP binding mode is analogous to that of other fold-type I aminotransferases, with the coenzyme engaging in contacts with highly conserved residues: Lys348, linked by a Schiff base to the aldehydic group of PLP, Phe210 and Pro297, generating pyridine ring stacking interactions, Ile262, which participates in a hydrophobic interaction with the C2′ atom of PLP, Tyr298 and Asn267, which form hydrogen bonds with the O3 atom, and Asp295, whose side chain in engaged in a salt bridge with the pyridine nitrogen. In addition, the phosphate group of PLP is stabilized by several polar contacts including hydrogen bonds with the main chain of Ser185 (Asn141 in Aro8) and Asn186 and with the side chains of Ser345, Ser347, and Tyr149^*^ (^*^denoting a residue from the neighboring subunit) as well as a salt bridge interaction with the guanidinium group of Arg355. Overall, these results well agree with spectroscopic data indicating that AroH displays a similar PLP binding mode as Aro8 and a similar pH-dependence of the internal aldimine (Figure 2; Karsten et al., 2011).

**Figure 8:**
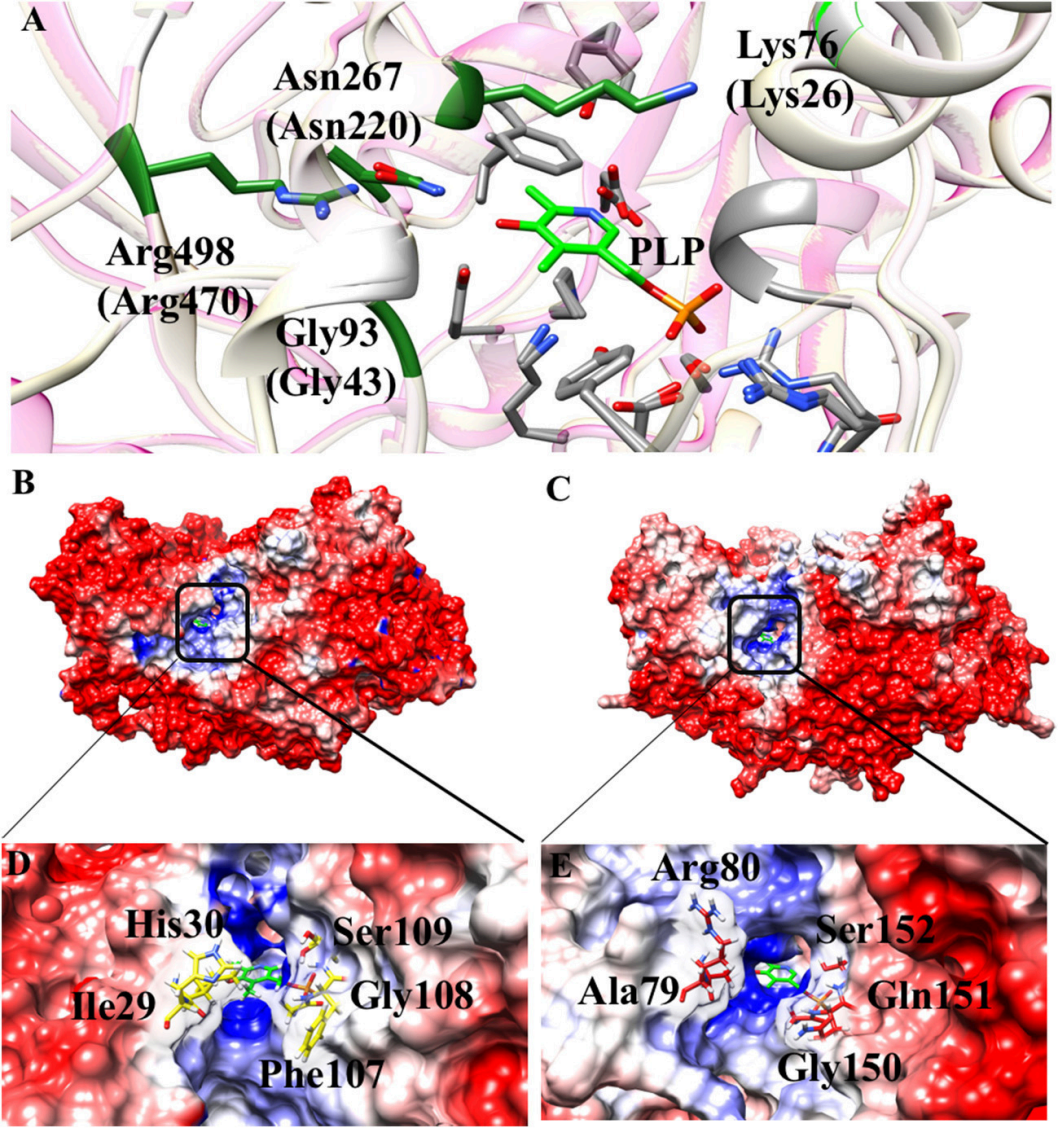
Structural features of the AroH active site regions. **(A)** Ribbon representation of superimposition of the region surrounding the active site in AroH and Aro8. Residues involved in PLP or substrate binding are highlighted as gray or green sticks, respectively. The PLP cofactor is shown as light green stick. **(B,C)** Electrostatic surface potential of Aro8 **(B)** and AroH **(C)** calculated using non-linear Poisson Boltzmann equation [−4 (red) and +4 (blue) kT/e] at 25°C, pH 7.4. **(D,E)** Particular of the electrostatic map of the active site channel of Aro8 **(D)** and AroH **(E)**. Residues involved in the formation of active site channel of Aro8 are highlighted as sticks. Around the sticks transparency (80%) of the surface representation has been applied. Potential contours are [−4 (red) and +4 (blue) kT/e] around both proteins.

Some interesting observations can be also made by looking to regions surrounding the active site. In particular, in Aro8 it has been reported that Lys26, Gly43, Asn220, and Arg470 play an important role in substrate binding and recognition, as demonstrated also in other two enzymes of the same family (α-aminoadipate aminotransferase from *Thermus thermophilus* and hKAT II). In the AroH homology model, these residues (Lys76, Gly93, Asn267, and Arg498) are the same reported for Aro8 (Figure 8A), a feature possibly related to their preference for aromatic substrates. Moreover, Lys76 (corresponding to Lys26 in Aro8) in hKATII is replaced by Arg20, which participates in cation-π stacking with L-kynurenine mediating an important substrate binding interaction, and also plays a role in L-glutamate binding. The presence of a lysine instead of an arginine residue in AroH could at least partly explain its reduced catalytic efficiency for L-kynurenine transamination, as well as the increased value of the K_m_ for L-glutamate with respect to hKATII (Han et al., 2008; Ouchi et al., 2009).

Notwithstanding the similar active site topology between Aro8 and AroH, we noticed that some differences exist related to the physical-chemical features of the channel at the entrance of the active site pocket. As reported in Figures 8A–C, the surface of the two proteins has mainly a negative potential, while the active site region is composed by positively charged residues. However, arginine residues present in the surface of AroH decrease the negative potential at the entrance of the active site channel. Moreover, the residues Phe107, Ile31, and His30 of Aro8 are substituted by Gln151, Ala79, and Arg80, respectively, in AroH (Figures 8D–E). This increases the size of active site channel entrance in AroH, probably explaining the subtle differences in substrate specificity between the two proteins. In particular, a wider channel could allow the entrance of bulky substrates, such as L-tryptophan (see above), in line with the proposed role of AroH in the catabolism of this amino acid (Choera et al., 2017).

## Conclusions

In this work we have purified the recombinant form of *Aspergillus* AroH, a putative aromatic aminotransferase, and examined the biochemical features of the protein. Our spectroscopic results, combined with *in silico* modeling, indicate that AroH displays structural properties and PLP binding mode very similar to those of the yeast ortholog Aro8. We also setup a new enzymatic assay providing a rapid and reproducible measure of the transaminase activity, which could be easily implemented for high-throughput studies. By using this assay, we demonstrated the high kinetic versatility of AroH, which allows us to propose an involvement of the enzyme in both aromatic amino acids catabolism, and L-lysine biosynthesis. We have already shown that aminotransferases affects bacterial behavior and capacity to interact with the host immune system (Zelante et al., 2013). Should future studies point to an important functional role of fungal aminotransferases at the host/fungus interface, the results of the present study may be exploited for drug discovery of specific AroH modulators as innovative therapies targeting fungal metabolism.

## Author contributions

MD performed and designed the experiments. EC performed AroH purification. MP, GA, and AB contributed to *in silico* analyses. MP, CC, GA, AB, and NK critically read, analyzed, and discussed the literature and conceived the outline of the manuscript. LR and TZ wrote the manuscript, BC wrote the manuscript and supervised the project. All the authors edited the manuscript and provided valuable discussions and criticisms.

### Conflict of interest statement

The authors declare that the research was conducted in the absence of any commercial or financial relationships that could be construed as a potential conflict of interest.

## Abbreviations

PLP: pyridoxal 5'-phosphate
APAD^+^: 3-Acetylpyridine adenine dinucleotide
GDH: L-glutamate dehydrogenase
LDH: L-lactic dehydrogenase
KP: potassium phosphate buffer
SEC: size-exclusion chromatography
K_D(PLP)_: equilibrium dissociation constant for PLP
PMP: pyridoxamine 5'-phosphate
hKATII: human kynurenine aminotransferase II.
