# Supplementary material for: Biochemical Characterization of Aspergillus fumigatus AroH, a Putative Aromatic Amino Acid Aminotransferase
Source: Front Mol Biosci. 2018 Nov 28;5:104. doi: 10.3389/fmolb.2018.00104 (PMC6279937; doi:10.3389/fmolb.2018.00104)
Supplement: Supplementary file 1 [file Data_Sheet_1.docx]

**Biochemical characterization of *Aspergillus fumigatus* AroH, a putative aromatic amino acid aminotransferase**

**SUPPLEMENTARY INFORMATION**

**
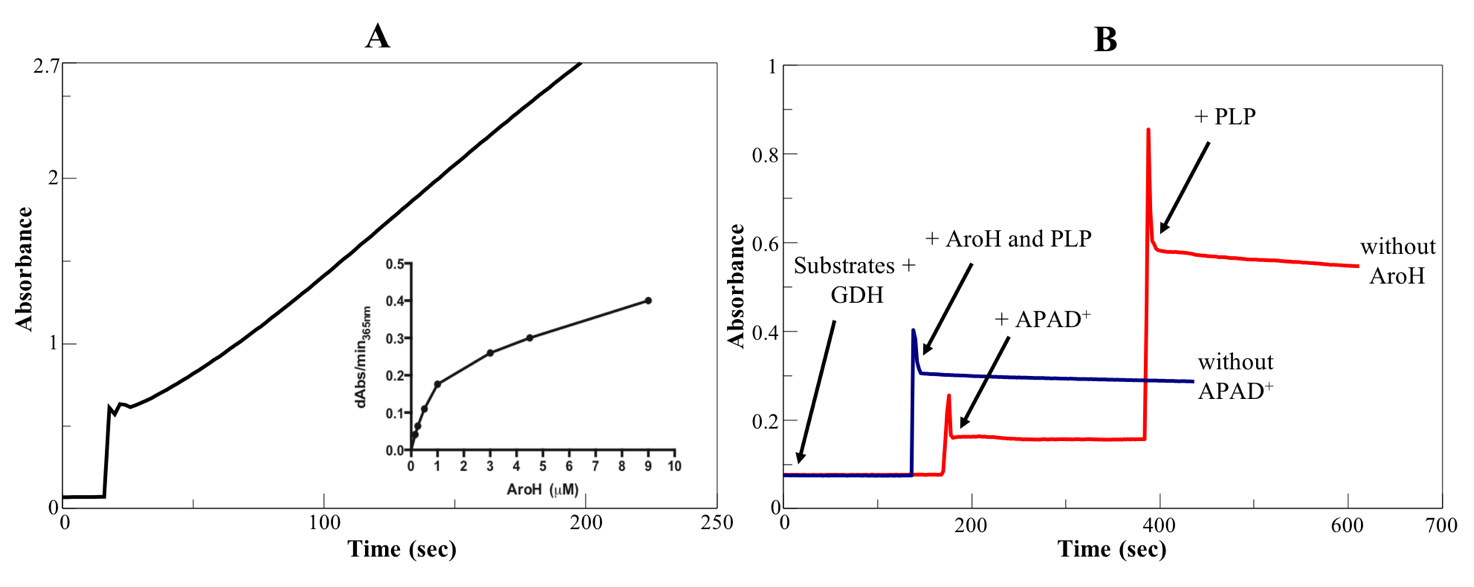
**

**Fig. 1S. AroH representative transaminase activity assay and negative controls. A)** Time-dependent changes of the absorbance at 365 nm during the continuous assay of AroH (0.5 μM) transaminase activity in the presence of 20 mM L-tryptophan and 1 mM α-ketoglutarate in 66 mM KP, pH 7.4 at 25°C. The inset shows the reaction rate as a function of AroH concentration. **B)** Assay negative controls without APAD^+^ (blue line) or without AroH (red line).

200

1500

500

1000

25

85

40

60

80

**Relative Fluorescence**

**Temperature (°C)**

**Fig. 2S. Thermal unfolding of AroH in the holo- and apo- form.** Intrinsic fluorescence emission was recorded at 1 μM apo- and holo-AroH (in the presence of 50 μM PLP) using λ_ecc_ at 280 nm and λ_em_ at 330 and 332 nm for holo (red line) and apo (blue line) AroH, respectively. The fluorescence emission of the cofactor has been recorder using λ_ecc_ at 350 and λ_em_ at 420 nm. The experiments have been performed in triplicate in KP 0.1 M pH 7.4.


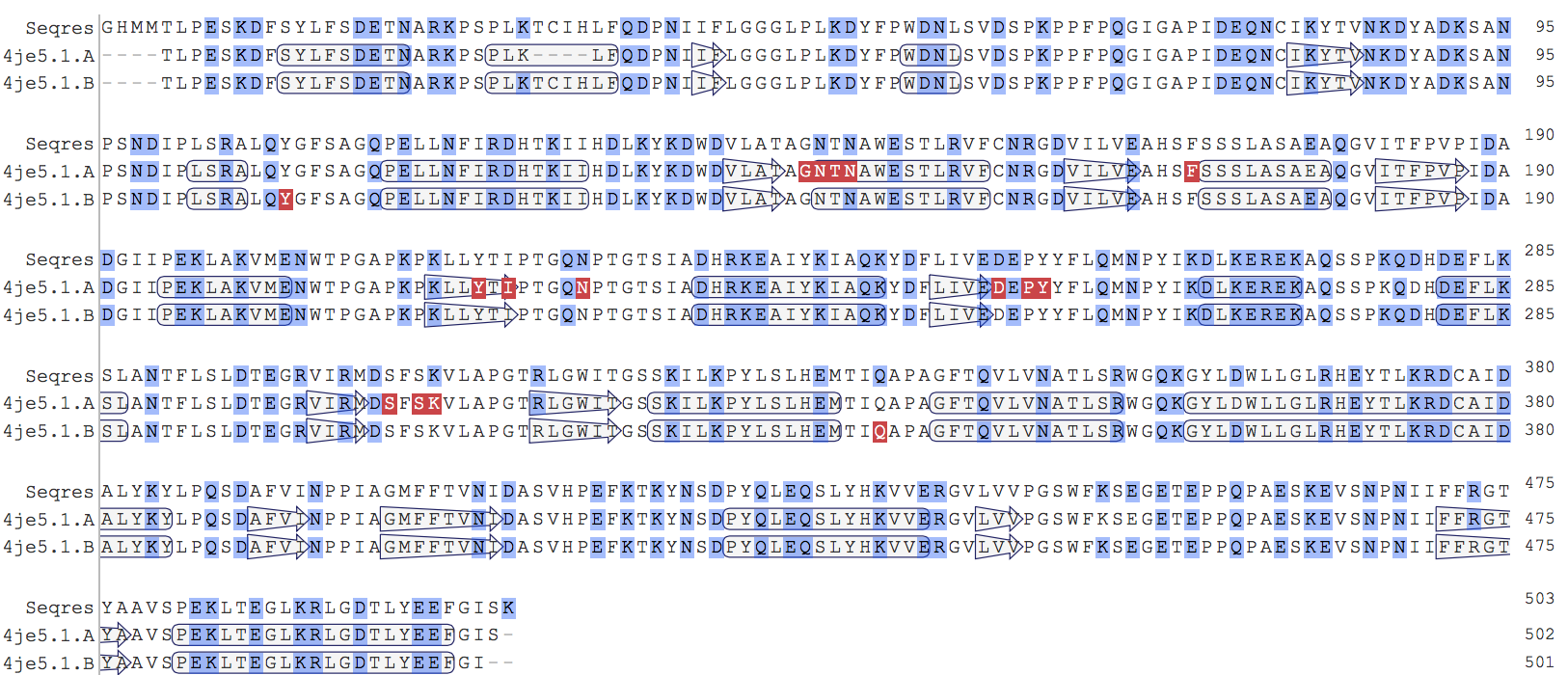


**Fig. 3S. Sequence alignment of AroH from *A. fumigatus* and subunits A and B of Aro8 from *S. cerevisiae***. Polar residues are highlighted in blue while residues involved in PLP binding in Aro8 are highlighted in red.


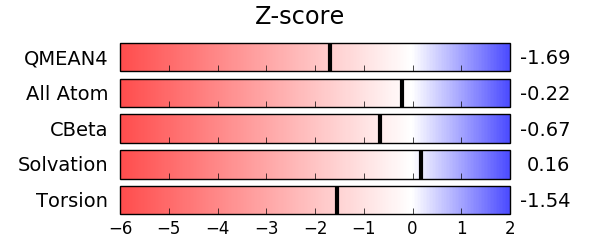

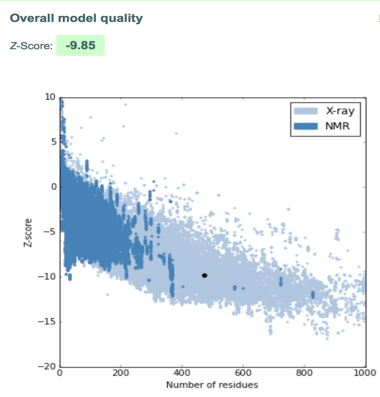

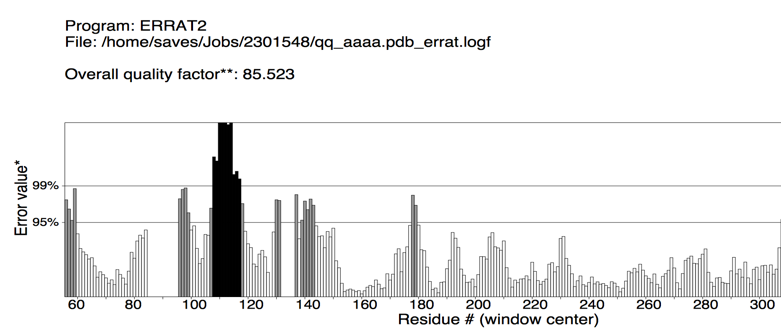

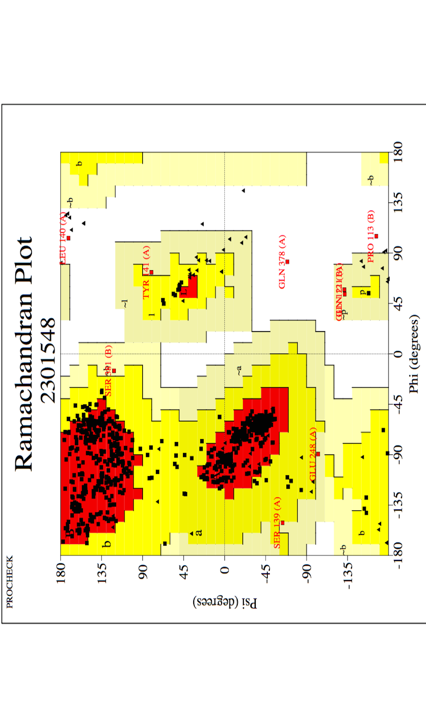


**A**

**B**

**D**

**C**

**Fig.4S. AroH structural model validation. Panel A**: QMEAN score obtained on the SWISS-PROT portal. **Panel B**: Z-score obtained using ProSA. **Panel C**: Overall quality factor obtained using ERRAT2. **Panel D:** Ramachandran plot calculated by PROCHECK.
